# Vaccine schedule recommendations and updates for patients with hematologic malignancy post‐hematopoietic cell transplant or CAR T‐cell therapy

**DOI:** 10.1111/tid.14109

**Published:** 2023-07-29

**Authors:** Gemma Reynolds, Victoria G. Hall, Benjamin W. Teh

**Affiliations:** ^1^ Sir Peter MacCallum Department of Oncology University of Melbourne Parkville Victoria Australia; ^2^ Department of Infectious Diseases Peter MacCallum Cancer Centre Melbourne Victoria Australia; ^3^ Department of Infectious Diseases Austin Health Heidelberg Victoria Australia

**Keywords:** bispecific, CAR‐T cell, cellular therapy, hematologic malignancy, stem cell transplant, vaccination

## Abstract

Revaccination after receipt of a hematopoietic cell transplant (HCT) or cellular therapies is a pillar of patient supportive care, with the potential to reduce morbidity and mortality linked to vaccine‐preventable infections. This review synthesizes national, international, and expert consensus vaccination schedules post‐HCT and presents evidence regarding the efficacy of newer vaccine formulations for pneumococcus, recombinant zoster vaccine, and coronavirus disease 2019 in patients with hematological malignancy. Revaccination post‐cellular therapies are less well defined. This review highlights important considerations around poor vaccine response, seroprevalence preservation after cellular therapies, and the optimal timing of revaccination. Future research should assess the immunogenicity and real‐world effectiveness of new vaccine formulations and/or vaccine schedules in patients post‐HCT and cellular therapy, including analysis of vaccine response that relates to the target of cellular therapies.

**Abbreviations**
aRZV: adjuvanted recombinant zoster vaccine
BCMA: B‐cell maturation antigen
CAR‐T: chimeric antigen receptor T cell
CD19: cluster of differentiation 19
CD20: cluster of differentiation 20
COVID‐19: coronavirus disease 2019
GvHD: graft‐versus‐host disease
HCT: hematopoietic cell transplant
HM: hematologic malignancy
IgG: immunoglobulin G
IVIg: intravenous immunoglobulin
MMR: measles, mumps, and rubella
PCV: pneumococcal conjugate vaccine
PPSV23: pneumococcal 23‐valent polysaccharide vaccine
SARS‐CoV‐2: severe acute respiratory syndrome coronavirus 2
VZV: varicella zoster virus

## 1 INTRODUCTION

**FIGURE 1:**
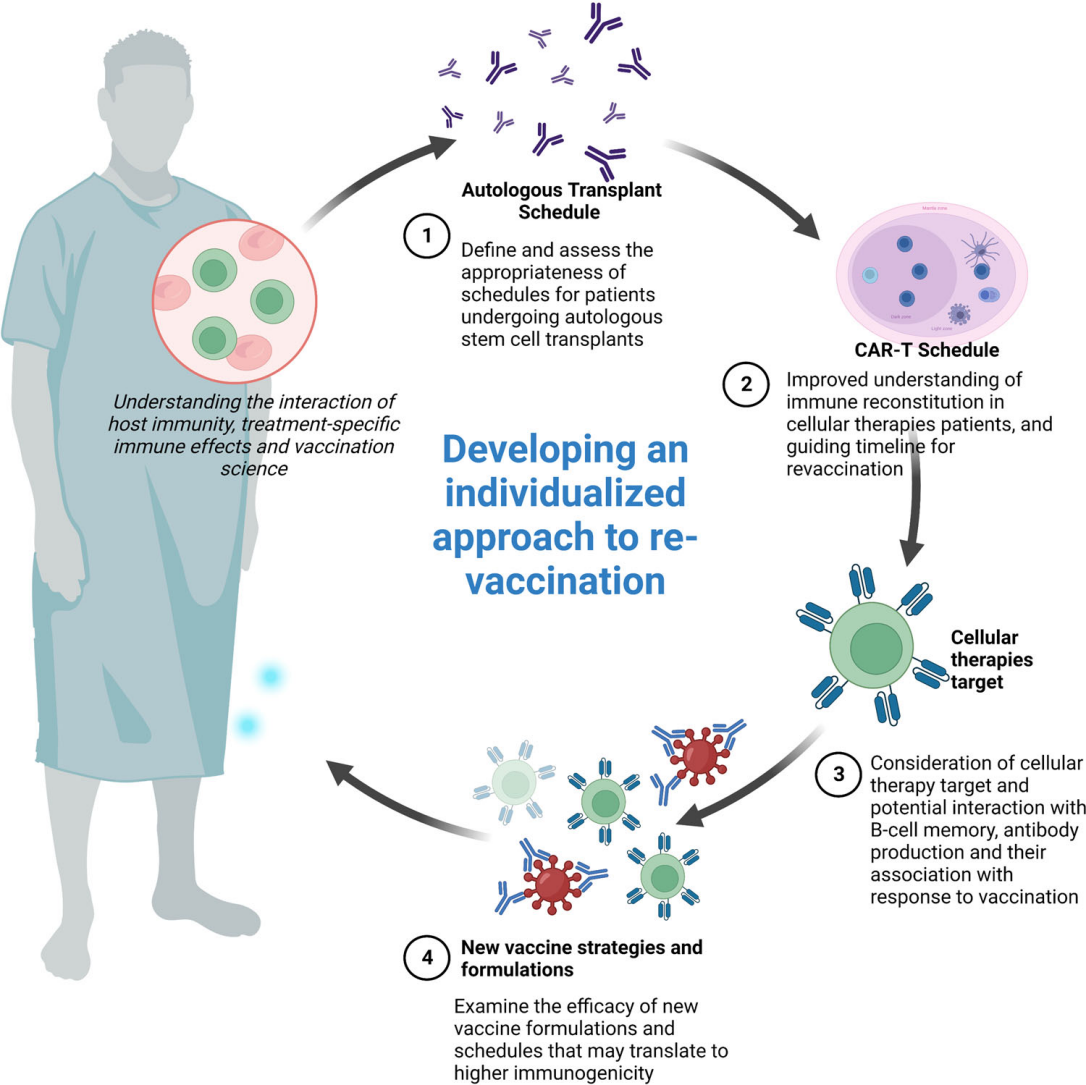
Future approaches to vaccine research for hematopoietic cell transplant (HCT) and cellular therapies patients.

Between 50 000 and 100 000 hematopoietic cell transplants (HCT) are performed annually worldwide.^1^, ^2^ Over 20 000 people globally have received Food and Drug Administration‐approved cellular therapy, particularly chimeric antigen receptor T‐cell (CAR‐T) product for treatment of lymphoma, myeloma, or leukemia.^3^ Infection remains a significant cause of morbidity and mortality among patients with hematological malignancies (HM), including those experiencing enduring remission after transplant or cellular therapies. Vaccination strategy represents a pillar of supportive care that can help augment protection against infection (Figure 1).

Together with antimicrobial prophylaxis, intravenous immunoglobulin (IVIg), and early antimicrobial therapy, vaccination aims to reduce the burden of infection in patients post‐transplant and cellular therapies. Regarding vaccine‐preventable infections, late viral reactivations, such as varicella zoster, are common among both HCT recipients and cellular therapy patients, with reports of severe disseminated disease.^4^, ^5^, ^6^, ^7^, ^8^ Similarly, instances of overwhelming sepsis secondary to encapsulated organisms, such as *Streptococcus pneumoniae* or *Haemophilus influenzae*, have been reported.^9^, ^10^, ^11^ The coronavirus disease 2019 (COVID‐19) pandemic demonstrated how hematology patients, especially those treated with cellular therapies and HCT, are disproportionately affected by infectious diseases that lack adequate prevention strategies, leading to more severe disease, more frequent re‐infections and poorer vaccine response.^12^, ^13^, ^14^, ^15^

However, marrying vaccine studies with clinical outcomes remains challenging. The clinical relevance of reduced antibody titers post‐HCT or cellular therapies is poorly understood, given that only a limited number of vaccine‐preventable diseases have been reported. For the general population, with exception of the influenza vaccine, there is a lack of immune correlates of absolute or relative protection for other vaccine‐preventable diseases, including COVID‐19.^16^, ^17^, ^18^, ^19^ When interpreting results from published studies of vaccine response in HCT recipients, it is important to recognize the dominant focus of humoral (serologic) endpoints, the lack of measurement of cell‐mediated responses and the lack of correlation between immunogenicity and clinical endpoints.^20^, ^21^ An ongoing challenge that remains, including recent examples of vaccination against Respiratory syncytial virus (RSV)^22^ and influenza,^23^ is that the inclusion of HCT recipients in initial clinical trials is often delayed despite being one of the highest risk groups for disease and related complications.

This review aims to comprehensively discuss the evidence for vaccination strategies in HCT patients, focusing on newer vaccine formulations, evidence for optimized dosing regimens, and vaccine adjuncts. It also aims to review the emerging evidence informing vaccination strategies following cellular therapies, highlighting questions around retained seroprevalence, vaccine response, and the interplay between immune reconstitution and vaccine timing. Potential future directions for translational vaccinology, including the individualization of revaccination approach in hematology patients, are discussed.

## 2 VACCINATION IN HCT RECIPIENTS

Infection after HCT is a major cause of morbidity and mortality, with vaccine‐preventable infections being a potential contributor. Underpinning this are complex deficiencies in humoral and cell‐mediated immune responses, including opsonization, related to the transplant conditioning regimen, hypogammaglobulinemia, prophylaxis or management of graft‐versus‐host disease (GvHD) for allogeneic HCT or maintenance therapies for autoHCT.^24^, ^25^, ^26^, ^27^, ^28^, ^29^, ^30^ Regardless of pre‐transplant donor or recipient vaccinations, HCT recipients may lose their immunity to various pathogens if the recipient is not revaccinated.^25^, ^26^, ^27^, ^28^, ^29^ Therefore, it is recommended to consider HCT patients as “never vaccinated” and requiring a full revaccination schedule, considering age, country, and epidemiology.

International guidelines with recommended schedules have been published, with commencement of vaccination usually occurring 3–6 months after HCT.^24^, ^31^, ^32^, ^33^, ^34^, ^35^, ^36^ This may differ between centers and be deferred (other than inactivated influenza vaccine) to commence at 12 months if there is potential for delayed immune reconstitution or negative impact of therapy, indicated by therapies received including anti‐cluster of differentiation 20 (CD20) mAb <6 months ago or blood parameters including CD4+ T‐cell count <200/μL and cluster of differentiation 19 (CD19)+ B‐cell count <20/μL.^24^, ^31^, ^32^, ^33^, ^34^, ^35^ There are no data to support any specific lymphocyte level for starting vaccines, however, and delaying vaccination increases the at‐risk period for the patient.^32^ After HCT, it may be reasonable to delay vaccination for at least 6 months after the last dose of anti‐CD20 mAb therapy and/or other B‐cell‐depleting therapies.^24^, ^32^

Inactivated vaccines are safe after transplant, although immunogenicity may be reduced post‐transplant compared with healthy individuals, with improvement over time and close to expected response at 2–3 years post‐transplant.^31^, ^37^ Inactivated vaccines are not inhibited by IVIg replacement.^24^ Patients with GvHD are at higher risk of infection and therefore benefit from vaccination; however, vaccine response may be impaired due to immunosuppression used to treat GvHD. Live‐attenuated vaccines are only recommended in specific circumstances and at least 24 months post‐transplantation due to the risk of vaccine‐transmitted disease.^31^, ^32^, ^33^, ^34^ Optimal vaccination of household contacts is encouraged, including live‐attenuated measles, mumps, and rubella (MMR) and varicella zoster virus (VZV) vaccine if indicated, but not intranasal live‐attenuated influenza vaccine or live‐attenuated oral polio vaccine due to prolonged viral shedding and risk of transmission.^33^, ^38^

Where available, updates are discussed in the sections below with existing recommendations by various international bodies summarized in Table 1.^31^, ^32^, ^33^, ^34^, ^35^, ^36^

**TABLE 1:** Recommended vaccines and timing post‐hematopoietic cell transplant (HCT) from established international guidelines.

Vaccine‐preventable infection and vaccine(s)	Recommendations						
	ECIL‐7 (2017)^32^	DGHO (2018)^141^	IDSA (2013)^33^	International (2009)^35^	UK (2023)^36^	ACIP (2023)^142^	AIH (2023)^143^
Pneumococcal: PCV13, PCV15, PCV20 PPSV23	Three doses of PCV 1 month apart from 3–6 months after HCT, followed by a fourth dose of PCV if GvHD or PPSV23 6 months later^32^				Three doses of PCV 1 month apart, from 3 to 6 months after HCT Booster dose given at 18 months post‐HCT with PCV if GvHD or PPSV23 if no GvHD	Three doses of PCV 1–2 months apart, from 3 to 6 months after HCT, with PPSV23 8 weeks after last dose of PCV or at 12 months post‐HCT. PCV as fourth dose if GvHD. New proposal to substitute fourth dose of PPSV23 with PCV20^144^	Three doses of PCV from 6 months after HCT at 6, 8, and 12 months post‐HCT PPSV23 starting at 24 months post‐HCT then >5 years later
Hemophilus type B: Hib conjugate vaccine	Three doses of Hib vaccine at 1‐month intervals, from 3 months after HCT OR Three doses of combined diphtheria–tetanus–pertussis–Hib vaccine from 6 months after HCT	Three doses of Hib vaccine, from 6 to 12 months post‐HCT		Three doses of HiB at 2‐month intervals, from 6 to 12 months post‐HCT	Similar to DGHO/IDSA	Three doses of Hib vaccine, at least 1‐month interval, at 6 months post‐HCT	Three doses of Hib vaccine at 6, 8, and 12 months after HCT
Neisseria meningitidis: Quadrivalent (ACYW135) Conjugate (MCV‐4) Monovalent C conjugate (MCV‐C) Anti‐B vaccines MenB‐MC MenB‐fHBP	Recommended in accordance with local guidelines Two doses of vaccine against serotypes B and C, 6 months post‐HCT	1–2 doses of conjugate tetravalent vaccine, 6–12 months post‐HCT	Two doses of MCV‐4 6–12 months post‐HCT for 11– 18 years	Follow country guidelines, one dose, 6–12 months post‐HCT	Two doses of MCV‐4, 8 and 10 months post‐HCT Two doses of a MenB vaccine at a 2‐month interval, at 6 months post‐HCT	MCV‐4 for individuals 11–18 years or at high risk from 6 months post‐HCT Serogroup B meningococcal vaccine for individuals 16–23 years of age or at high risk, at 6 months post‐HCT	Two doses of any MCV‐4 vaccine at 2‐month intervals, from 6 months post‐HCT MenB: Two doses of Bexsero, at 2 month intervals, from 6 months post‐HCT OR Three doses of Trumenba at 1 and 5‐month intervals, from 6 months post‐HCT
Diphtheria and tetanus^a^: DT (full dose diphtheria) Td (reduced dose diphtheria) DTaP (higher dose of tetanus, diphtheria, pertussis toxoid) Tdap (lower dose formulation) Bordetella pertussis^a^: Acellular pertussis vaccine—ap (pertussis toxoid)	Three doses of diphtheria–tetanus vaccine at 1–2‐month interval, from 6 months after HCT for all ages^145^ Full dose diphtheria (DT) vaccine preferred over reduced dose antigen formulation diphtheria toxoid (Td) to achieve adequate seroprotection Three doses (for pertussis) given in combination with each dose of the diphtheria–tetanus vaccine from 6 months after HCT					Three doses of DTaP for patients <7 years, 1–2‐month intervals, at 6 months post‐HCT For patients ≥7 years, options include: three doses of DTaP OR one dose of Tdap and two doses of DT OR one dose of Tdap and two doses of Td at 1–2‐month intervals, from 6 months post‐HCT	Three doses of diphtheria‐tetanus vaccine at 1–2 months interval, from 6 months after HCT DTPa for children <10 years dTpa for ≥10 years of age for first dose then two doses of dT. If unavailable, then complete with dTpa
Polio virus^a^: IPV DTPa‐IPV dTpa‐IPV	Three doses of IPV administered at 1–2‐month intervals, 6–12 months after HCT						Three doses of IPV administered at 1–2‐month intervals 6–12 months after HCT Can be given in combination with diphtheria, tetanus, pertussis vaccines
HPV: 4vHPV or 9vHPV	Follow local guidelines for general population and start 6–12 months post‐HCT				Three‐dose schedule is recommended for all HCT recipients aged 12 and over ACIP currently advises vaccination with either 4vHPV or 9vHPV in immunocompromised persons age 9–26 years		Three doses of 9vHPV at 0, 2, 6 months, starting at 8 months post‐HCT If >25 years of age, conduct risk assessment to determine their need for HPV vaccination
HBV: HBV vaccine Engerix	Three doses at 0, 1, and 6 months apart, 6–12 months post‐HCT in those non‐immune to HBV		Three doses at 0, 1, and 6 months apart at 6–12 months post‐HCT	Follow general population recommendations in their country of residence	Three doses at 0, 1, and 6 months, from 6 months post‐HCT	Three doses at 0, 1, and 6 months apart, from 6 months post‐HCT	Three doses (high‐dose formulation is preferred) at 6, 8, and 12 months post‐HCT
Influenza virus: IIV Live intranasal influenza vaccine is contraindicated	One dose of IIV, annually, at the beginning of influenza season Second dose at 3–4 weeks later could be considered in patients expected to have impaired immune response, or during outbreaks in patients vaccinated <6 months post‐HCT	One dose of IIV, annually, at 3–6 months post‐HCT Second dose should be considered in patients who have had early vaccination	One dose of IIV annually, starting 6 months post‐HCT or 4 months post‐HCT if there is a community outbreak	One dose of IIV annually Consider second dose if the vaccine is given <6 months post‐HCT	One dose of IIV annually, from 6 months post‐HCT Consider commencing at 3 months post‐HCT if a peak influenza transmission period	One dose of IIV, annually from 6 months post‐HCT Second dose if first dose is given <4 months after HCT	Two doses of IIV in the first year post‐HCT and then one dose annually thereafter. Adjuvant IIV recommended for those ≥65 years of age^143^
SARS‐CoV‐2	Predated SARS‐CoV‐2				Three doses of COVID‐19 (preferably mRNA) vaccine starting 3–6 months post‐HCT as primary course Further booster vaccine doses recommended as per local guidelines, and at least 3–6 months after the last dose of vaccine^36^, ^75^, ^79^, ^146^		
Varicella zoster virus: LAVV	LAVV can be administered at ≥24 months post‐HCT in varicella‐seronegative HCT recipients who do not have active GvHD, are not receiving any immunosuppression (some recommend for >12 months) and are 8–11 months after the last dose of IVIg (UK guidelines suggest 3 months post‐IVIg)^24^, ^33^, ^48^, ^49^, ^50^ A second dose could be administered 4 weeks after the first					LAVV at 24 months post‐HCT and no GvHD and the patient is considered immunocompetent	Two doses of LAVV, 4 weeks apart at 24 months post‐HCT if there is evidence of immune reconstitution
Herpes zoster virus: aRZV, adjuvanted recombinant zoster virus vaccine (inactivated), Shingrix	Predated aRZV vaccine				Two doses aRZV at least 2 months apart, commencing at 6 months following HCT	Two doses of aRZV 6–12 months post‐allogeneic SCT and 3–12 months post‐autologous SCT	Two doses of aRZV at 6 and 8 months post‐HCT
MMR: MMR live virus vaccine	One dose of MMR vaccine to seronegative HCT recipients >24 months post‐transplantation; no GvHD, no immunosuppression, no relapse of the underlying disease, no recent IVIg (at least 3 months)		Two doses of MMR vaccine to seronegative patients with same criteria applied		Two doses of MMR vaccine, 6 months apart in adult recipients of autologous and allogeneic HCT who are 24 months post procedure, no GvHD, no systemic immunosuppression for >12 months, and are seronegative	MMR vaccine at 24 months post‐HCT if the patient does not have GvHD and is considered immunocompetent	One dose of MMR vaccine at 24 months post‐HCT if they have met criteria. Check serology at 4–6 weeks after 1st vaccine dose—if no seroconversion, repeat the dose

Abbreviations: 4vHPV, 4‐valent human papillomavirus vaccine; 9vHPV, 9‐valent human papillomavirus vaccine; ACIP, Advisory Committee on Immunization Practices; ACYW‐135, meningococcal quadrivalent conjugate vaccine; AIH, Australian Immunization Handbook; ap, acellular pertussis vaccine; aRZV, adjuvanted recombinant zoster virus vaccine; COVID‐19, coronavirus disease 2019; DGHO, German Society of Hematology and Oncology; DT, full dose diphtheria vaccine; DTaP, higher dose diphtheria, tetanus, and acellular pertussis vaccine; DTPa‐IPV, higher dose diphtheria, tetanus, acellular pertussis, and inactivated poliovirus combination vaccine; dTpa‐IPV, reduced antigen formulation diphtheria–tetanus–pertussis–inactivated poliovirus combination vaccine; ECIL‐7, European Conference on Infections in Leukaemia; GvHD, graft‐versus‐host disease; HBV vaccine, hepatitis B virus; Hib, *Haemophilus influenzae* type B conjugate vaccine; HiB, *Haemophilus influenzae* B; HPV, human papillomavirus vaccine; IDSA, Infectious Diseases Society of America; IIV, inactivated influenza vaccine; IPV, inactivated polio virus vaccine; IVIg, intravenous immunoglobulin; LAVV, live‐attenuated varicella vaccine; MCV‐4, meningococcal conjugate vaccine; MCV‐C, monovalent meningococcal serogroup C conjugate vaccine; MenB‐fHBP—Trumenba, recombinant lipidated factor H binding protein meningococcal serogroup B vaccine; MenB‐MC, recombinant multicomponent meningococcal serogroup B vaccine; MMR, measles, mumps, and rubella live virus vaccine; PCV, pneumococcal conjugate vaccine; PPSV23, pneumococcal 23‐valent polysaccharide vaccine; SARS‐CoV‐2, severe acute respiratory syndrome coronavirus 2; Td, reduced dose diphtheria–tetanus combination vaccine; Tdap, reduced dose tetanus, diphtheria, acellular pertussis combination vaccine.

^a^ Combination vaccine available diphtheria–tetanus–pertussis plus polio (dTpa‐IPV).

### 2.1 Updated or new formulation vaccines for HCT

#### 2.1.1 Streptococcus pneumoniae

There is a recognized high risk of invasive pneumococcal disease after autologous and allogeneic HCT, related to low specific antibody titers, with approximately 85% of patients unprotected 6 months after transplantation.^10^, ^39^, ^40^ Of note, most patients are infected by vaccine serotypes.^9^, ^41^ The current guideline recommendation is to provide three doses of pneumococcal conjugate vaccine (PCV) 1 month apart starting 3–6 months post‐HCT. The increased immunogenicity and clinical effectiveness of PCV compared to pneumococcal 23‐valent polysaccharide vaccine (PPSV23) has been demonstrated.^30^, ^32^, ^33^, ^42^, ^43^, ^44^, ^45^ This is then followed by a fourth dose of PCV if the patient has GvHD or one dose of PPSV23 6 months later to increase the spectrum of serotype coverage.^30^, ^32^, ^33^ Some centers may start pneumococcal vaccination at 12 months post‐HCT and check serology 1 month after the third or fourth dose of vaccine.

Newer vaccine formulations are now available in some countries, including 20‐valent PCV (PCV20) and 15‐valent PCV (PCV15), offering additional serotype coverage. There are no current studies of PCV20 in HCT recipients. An alternate strategy proposed by the Advisory Committee on Immunization Practices is to provide a fourth dose of PCV20 (in place of PPSV23) at least 6 months after the third PCV20 dose or 12 months after HCT, whichever is later. This specific recommendation has not been studied; however, it is based on two studies that assessed the use of four PCV13 doses and found improved humoral immunity from after the third dose to after the fourth dose.^45^, ^46^ Local and systemic reactions however occurred more frequently after the fourth PCV13 dose than after the first to third dose of PCV13, although most were mild–moderate.^45^

#### 2.1.2 Varicella zoster virus vaccines

The prevention of primary VZV infection in seronegative patients and reactivation in seropositive patients (zoster and postherpetic neuralgia) is of prime importance in patients post‐HCT.^7^, ^8^ Antiviral prophylaxis (acyclovir or valacyclovir) is usually administered for at least 24 months post‐allogeneic HCT and 12 months after autologous HCT; however, may be longer if there is ongoing immunosuppression or GvHD.^7^, ^8^, ^47^ However, there are limitations to prophylaxis, including uncertainty around optimal duration, ongoing compliance, and lack of reconstitution of cellular immunity. Based on evidence from several observational studies, the live‐attenuated varicella vaccine can be administered at ≥24 months post‐HCT in varicella‐seronegative HCT recipients who do not have active GvHD, are not receiving any immunosuppression (some recommend for >12 months) and are not receiving immunoglobulin (Ig) replacement.^24^, ^33^, ^48^, ^49^, ^50^ There is an expected response rate of ∼65% with some clinical protection. A second dose could be administered 4 weeks after the first, although evidence of clear benefit is lacking.^24^, ^33^, ^48^, ^49^, ^50^ The risks and benefit of this decision should be carefully considered, given reports of fatal cases of vaccine‐related disseminated VZV.^6^, ^51^ Patients should be aware of this risk within 3 weeks of vaccination and to seek antiviral treatment if required.^32^ The live‐attenuated zoster vaccine (Zostavax) is not recommended by some published guidelines due to higher antigen levels in this formulation and has been associated with case reports of fatal disseminated vaccine‐strain infection.^32^, ^33^, ^35^, ^52^, ^53^, ^54^ However, Zostavax has been assessed in patients >24 months post‐HCT with adequate immunogenicity and no adverse effects on safety profiling in carefully selected patient cohorts.^55^, ^56^, ^57^, ^58^, ^59^ Zostavax is no longer marketed in the United States. An inactivated version of Zostavax was also studied in autologous HCT recipients and found to be efficacious against the prevention of herpes zoster; however, it has not been made commercially available.^60^

Shingrix, an inactivated, adjuvanted recombinant zoster vaccine (aRZV) is now available and recommended to prevent reactivation in seropositive immunocompromised patients.^61^, ^62^ It is a subunit vaccine containing VZV glycoprotein E and the AS01b adjuvant system.^5^, ^62^ In a large, international, multicenter phase III clinical trial, 1846 adult autologous HCT recipients were randomized to receive two doses of aRZV or placebo 50–70 days following HCT and then 1–2 months later.^5^ After a median of 21 months of follow‐up, the vaccinated group had a lower incidence of herpes zoster compared with the placebo group, with reported efficacy of 68.2%.^5^ Humoral immune response rates were in the range of 70%–80%, with similar rates reported in other immunocompromised patients, including those with HM, solid tumors, human immunodeficiency virus, and solid organ transplant.^5^ There was also reduction in postherpetic neuralgia, herpes zoster virus‐associated complications and hospitalization.^5^

For allogeneic seropositive HCT recipients, only observational data exists for the assessment of adjuvanted vaccines including the aRZV vaccine.^63^, ^64^ In these studies, patients have been vaccinated between 9 months to greater than 2 years post‐allogeneic HCT.^63^, ^64^, ^65^ There have been mixed results in regard to immunogenicity but no increase in GvHD.^63^, ^64^, ^65^ In one study, cell‐mediated immunity was significantly enhanced in patients with prior shingles compared to those without prior shingles and male sex.^65^ The recommended aRZV vaccine schedule is with two doses 2–6 months apart and to continue antiviral prophylaxis 1 month after the first vaccination. The use of aRZV in allogeneic HCT recipients and optimal timing need to be defined through future studies.

For all zoster vaccines, it is important to acknowledge that efficacy is difficult to assess, given that most of the data are based on company‐developed assays and are specific to these vaccine studies. Currently, there are no commercially available methods for addressing the protection of herpes zoster.

#### 2.1.3 COVID‐19 vaccines

Observational studies have demonstrated impaired humoral immune response post two doses of vaccine in patients post‐HCT, with estimated seroresponse rates of 61%–81%.^66^, ^67^, ^68^, ^69^ B‐cell‐depleting therapy within 12 months has been found to be a major determinant of impaired humoral immunity.^67^, ^70^, ^71^ Despite this, there does appear to be a preserved cellular immune response, even in patients treated with B‐cell‐depleting therapy, which has demonstrated prior importance in the severity of COVID‐19 infection.^66^, ^72^, ^73^, ^74^ In this context, the recommended primary course of vaccination is with three doses of COVID‐19 vaccine.^67^, ^75^, ^76^, ^77^, ^78^, ^79^, ^80^ Seroresponse rates post three doses of vaccine are improved compared to two doses and have been estimated to be in the range of 63%–90% in autologous HCT recipients and 58%–90% in allogeneic HCT recipients.^66^, ^81^, ^82^, ^83^, ^84^ This may be dependent on timing of vaccination relative to the transplant and use of any concurrent immunosuppressive therapies.^69^, ^77^ In allogeneic HCT recipients, lower seroresponse to three doses of vaccine has been found in HCT recipients of haploidentical donors, those with chronic kidney disease and lower lymphocyte count.^69^, ^73^ Starting from 3 to 6 months after transplantation, it is recommended to revaccinate HCT recipients with a full primary series of three doses, following the recommended schedule for each specific vaccine.^75^, ^79^ Immunogenicity analysis (humoral and cell‐mediated immunity) from a recent observational cohort study of allogeneic HCT recipients supports the commencement of mRNA severe acute respiratory syndrome coronavirus 2 (SARS‐CoV‐2) revaccination at 3 months post‐transplantation, immune responses were not influenced by concurrent GvHD or immunosuppressive regimen.^85^ The third dose should be given at least 28 days after the second dose. The mRNA vaccine alternatives (Pfizer Bio‐N‐Tech or Moderna mRNA‐1273) are the preferred option where available due to greater efficacy and safety evidence in immunocompromised patients for an expanded primary course and further booster doses.^67^, ^75^, ^76^, ^77^, ^78^, ^79^, ^86^ Booster dose(s) are recommended after the three‐dose primary course to optimize immunogenicity, and ^86^ the timing is suggested as per local guidelines but at least 3 months after the third dose.^76^ At the time of writing, updated variant‐specific mRNA COVID‐19 vaccines are now preferred as the booster dose.^79^

Clinical effectiveness studies have found reduced severity of COVID‐19 in vaccinated patients with HM; however, there is still significant morbidity and mortality from breakthrough infection.^13^, ^75^, ^87^ In a study of four‐dose vaccinated patients with HM, rates of hospitalization, oxygen requirement, and mortality of 38.2%, 21.6%, and 3.9%, respectively, were found with breakthrough COVID‐19.^87^ Waning immunity and reduced vaccine effectiveness against SARS‐CoV‐2 variants of concern have also been observed in patients with HM.^88^ Alternate vaccination strategies, including delayed intervals between vaccine doses, heterologous dosing, and bivalent Omicron‐specific mRNA vaccines, have shown improved immunogenicity in the general population and require ongoing assessment in HCT recipients.^89^, ^90^, ^91^, ^92^, ^93^, ^94^

Non‐mRNA COVID‐19 vaccines are also available, including non‐replicating competent adenovirus vector vaccines (AstraZeneca/ChAdOx1‐S and Johnson&Johnson/Jansen/Ad26.COV2‐S), as well as the recombinant nanoparticle protein‐based vaccine Novavax/NVX‐CoV2373, although published safety and efficacy data are limited in HCT recipients.^75^, ^95^, ^96^, ^97^ In a small study of hemato‐oncological patients, Ad26.COV2‐S appeared safe as a heterologous vaccine booster after two doses of BNT162b2 vaccine.^98^ In hematology malignancy patients with diseases and treatments impacting B‐cell immunity, in those who received two doses of ChAdOx1 vaccination followed by an mRNA vaccine, even in the absence of seroconversion robust SARS‐CoV‐2‐specific T‐cell immunity was documented.^74^

## 3 VACCINATION FOLLOWING CELLULAR THERAPIES

Less is known about the burden of vaccine‐preventable infections and the role of revaccination in patients treated with cellular therapies.^11^, ^99^, ^100^ Late‐infection data are still emerging, and the long‐term effects of sustained B‐cell depletion on infection are unclear.^101^, ^102^, ^103^ Prolonged B‐cell aplasia, loss of memory B cells, and subsequent hypogammaglobulinemia may impair humoral protection against various infections, including vaccine‐preventable diseases.^101^, ^104^, ^105^, ^106^, ^107^ The field is currently aiming to determine the extent to which antibody immunity is preserved following cellular therapies, optimize timing of revaccination following treatment, and identify key predictors of vaccine response from hematological markers of immune reconstitution.

### 3.1 Preservation of immunity: the retention of pathogen‐specific antibodies following cellular therapies

Few studies have systematically explored the persistence of pathogen‐specific IgGs after CAR‐T therapy. Available seroprevalence data is often adjunctive and exploratory; for example, comparing the prevalence of existing antibodies to vaccine‐preventable infections between CAR‐T treated patients and healthy controls to explore reasons for poor humoral response following SARS‐CoV‐2 vaccination.^108^, ^109^ Studies specifically aiming to characterize the effect of CAR‐T therapy on circulating pathogen‐specific IgGs are summarized in Table 2.^110^, ^111^, ^112^, ^113^, ^114^, ^115^ Studies differ in the timing of antibody measurement post‐CAR‐T, the exclusion or inclusion of patients receiving Ig replacement, and data are aggregated across mixed populations of leukemia, lymphoma, and myeloma patients.^110^, ^111^, ^112^, ^113^, ^114^, ^115^, ^116^ Data regarding seroprevalence in patients treated with bispecific or non‐CD19+ directed CAR‐T (e.g., B‐cell maturation antigen [BCMA]) therapies are lacking.^117^

**TABLE 2:** Summary of studies examining seroprevalence of circulating immunoglobulin G against vaccine‐preventable infections.

Author (year)	Angelidakis et al. (2022)^110^	Bansal et al. (2021)^111^	Rahman et al. (2019)^116^	Hill et al. (2019)^113^	Shah et al. (2021)^114^	Walti et al. (2021)^115^
Design	Prospective	Retrospective	Prospective	Retrospective	Retrospective	Retrospective
Population	38	40	8	40	21	85
Malignancy	Mixed	Lymphoma	DLBCL	Mixed	DLBCL	Mixed
CAR‐T target	CD19+	CD19+	CD19+	CD19+	CD19+	CD19+ BCMA
Prior HCT	5 (13)	25 (63)	Not reported	Not reported	5 (24)	32 (49)
IVIg	3/31 (10)	23 (58)	Not reported	Excluded	9 (43)	Excluded
Study baseline	Pre‐CAR‐T	Pre‐CAR‐T	Pre‐CAR‐T	Pre‐CAR‐T	12 months from CAR‐T	Median 20 months from CAR‐T
VZV	–	31/38 (82)	–	–	18 (87)	23/26 (90)
Hepatitis A	–	16/34 (47)	–	–	–	12/28 (41)
Hepatitis B	–	8/35 (23)	–	–	11 (52)	11/28 (40)
Measles	–	32/38 (84)	6/8 (75)	22/40 (55)	16 (77)	24/30 (80)
Mumps	–	10/12 (83)	5/8 (63)	–	18 (86)	15/30 (50)
Rubella	–	33/38 (87)	3/8 (38)	–	17 (81)	27/30 (90)
Pneumococcal	8/37 (22)	–	–	–	0 (0)	0/25
Tetanus	37/37 (100)	36/37 (97)	8/8 (100)	–	20 (95)	25/28 (90)
Diphtheria	35/37 (95)	–	7/8 (88)	–	–	25/28 (90)
Pertussis	–	–	0/8 (0)	–	11 (50)	0/14
Study endpoint	6 months post‐CAR‐T	3 months post‐CAR‐T	N/A	12 months post‐CAR‐T	N/A	N/A
VZV	–	26/27 (96)	–	–	–	–
Hepatitis A	–	22/31 (71)	–	–	–	–
Hepatitis B	–	22/31 (71)	–	–	–	–
Measles	–	22/27 (81)	6/8 (75)	21/40 (53)	–	–
Mumps	–	26/27 (96)	3/8 (63)	–	–	–
Rubella	–	26/27 (96	5/8 (38)	–	–	–
Pneumococcal	2/3 (67)	5/35 (14)	–	–	–	–
Tetanus	13/13 (100)	27/27 (100)	8/8 (100)	–	–	–
Diphtheria	11/13 (85)	–	7/8 (88)	–	–	–
Pertussis	–	–	0/8 (0)	–	–	–

Abbreviations: BCMA, B‐cell maturation antigen; CAR‐T, chimeric antigen receptor T cell; CD19, cluster of differentiation 19; DLBCL, diffuse large B‐cell lymphoma; HCT, hematopoietic cell transplant; IVIg, intravenous immunoglobulin; VZV, varicella zoster virus.

#### 3.1.1 The effects of CAR‐T target

Significant discussion surrounds how CAR‐T patients retain antibodies after B‐cell aplasia. One theory is that although CD19 directed CAR‐T cells deplete memory‐B and CD19+ plasma cells, they spare a CD19– plasma cell population, which replenish antibody production against previously encountered pathogens.^118^ BCMA CAR‐T, used in the treatment of multiple myeloma, depletes all BCMA‐expressing plasma cells and could therefore be associated with lower antibody positivity after treatment compared to the CD19+ product.^103^ At present, seroprevalence studies in BCMA treated patients are very limited, but a small study demonstrates (BCMA = 4) a lower rate of aggregated seropositivity (48%) compared to CD19+ treated patients (67%).^115^

#### 3.1.2 Streptococcus pneumoniae

Fewer than 14% of patients, not receiving IVIg replacement after CAR‐T, demonstrated antibodies against tested serovars of *S. pneumoniae* at 3–20 months post‐infusion.^114^, ^115^ These estimates reflect predominantly patients with lymphoma receiving CD19+ depletion. There is a lack of baseline data on *S. pneumoniae* seroprevalence prior to CAR‐T infusion.^110^ Similarly, reference data for healthy population seroprevalence for *S. pneumoniae* were not available.^115^ Small subgroups (*N* = 13) with *S. pneumoniae* IgG detected prior to CAR‐T infusion have demonstrated reduction in mean titers of IgG 3–6 months post‐infusion, although a proportion remained seroprotection throughout.^109^, ^110^

#### 3.1.3 Diphtheria, tetanus, and pertussis

Studies demonstrate a high rate of circulating IgG specific to tetanus (100% of patients) prior to CAR‐T infusion that was maintained, or comparable to healthy controls, at 3–20 months post‐infusion in 90%–100% of sampled patients.^108^, ^110^, ^111^, ^116^, ^119^, ^120^ Similarly, diphtheria antibodies were detected in 88%–95% of patients pre‐infusion, with similar seroprevalence maintained at post‐infusion follow‐up (median 3–20 months).^110^, ^112^ Pertussis data were sparse, with low seroprevalence rates identified at pre‐infusion in small samples, and equally low rates of detection at 12‐ and 20‐month follow‐up.^112^, ^114^, ^115^

#### 3.1.4 Measles, mumps, and rubella

Moderate seropositivity to measles (55%–84%), mumps (63%–83%), and rubella (38%–87%) was appreciated in CAR‐T patients prior to cellular therapy.^111^, ^112^, ^113^ If enduring response was achieved, patients maintained expression of MMR IgG.^111^, ^112^, ^114^, ^115^

#### 3.1.5 Varicella zoster virus

Within the limitations of a single study with pre‐infusion results, a high proportion of CAR‐T treated patients had demonstrable antibodies against VZV (82%).^111^ Post‐infusion serosurveys have demonstrated similar seroprevalence (87%–96%).^114^, ^115^ Seropositive patients are currently prophylaxed against VZV reactivation for between 6 and 12 months depending on center.^121^, ^122^, ^123^

### 3.2 Vaccine response

Most research examining vaccine response following CAR‐T therapies have focused on response to SARS‐CoV‐2 vaccines and are synthesized in existing systematic reviews.^81^, ^84^, ^124^, ^125^ Prospective observational studies have demonstrated that one or two vaccines are insufficient to elicit a robust humoral response in patients receiving cellular therapies, although the T‐cell response remains comparable to healthy controls.^108^, ^126^, ^127^, ^128^ Higher rates of antibody seroconversion are observed with subsequent booster doses (3–5 vaccines), and this is currently recommended HM patients, including those on cellular therapies.^36^, ^129^, ^130^, ^131^ Furthermore, mRNA vaccines elicited improved vaccine responses in this population compared to non‐mRNA vaccines.^125^ Predictors of vaccine response were heterogeneously modeled between studies. Steroid use and underlying non‐Hodgkin lymphoma predicted poor vaccine response.^84^, ^126^, ^132^ Higher numbers of circulating B cells were predictive of improved vaccine response in two studies.^129^, ^133^ Across studies, absolute lymphocyte count, Ig replacement, time since CAR‐T, and age of the patient were not predictive.^84^, ^132^, ^134^

Most vaccine studies have examined CD19+ directed CAR‐T therapies. Mechanistically, it is plausible that CD19+ and BCMA CAR‐T might differ in vaccine response.^135^ BCMA COVID‐19 vaccine data are again scarce and driven predominantly by a single study (*N* = 19) in which 79% of patients demonstrated seroconversion to SARS‐CoV‐2 after two doses.^136^, ^137^ The prior treatment history, time between CAR‐T therapy and vaccination, presence or absence of IVIg replacement, and levels of circulating B lymphocytes in this cohort are not known.

To date, only two studies have examined vaccine responses outside of COVID‐19 in CAR‐T treated patients. Lee et al.^109^ retrospectively examined paired pre‐ and post‐vaccine IgG titers in response to PCV13 vaccination in a cohort of CAR‐T treated lymphoma patients. Patients vaccinated at either day 90 (*N* = 13) or day 180 (*N* = 8) post‐CAR‐T demonstrated a reduction in titers between pre‐vaccination baseline and 90 days post‐vaccine.^109^ Of the eight patients in enduring remission at day 540 who demonstrated seroprotection against pneumococcus, three had confirmed baseline immunity prior to CAR‐T infusion, a further three patients were vaccinated, one patient received IVIg, and one patient received neither IVIg nor vaccination but had an unknown baseline antibody response.^109^

Additionally, Walti et al.^117^ prospectively examined response to the quadrivalent influenza vaccine in a mixed lymphoma/myeloma population treated with CD19+ and BCMA directed CAR‐T. Partial response to inactivated influenza vaccine was appreciated in 60%–70% of the cohort, defined as a twofold increase in antibody response to a vaccine strain, while 31%–40% of the cohort had robust antibody response (fourfold increase).^117^ There was no association between vaccine response and age, time from CAR‐T, IgG level, or underlying malignancy.^117^

### 3.3 Vaccination guidelines

Vaccine studies in patients undergoing treatment with cellular therapies remain limited. Thus, vaccination schedules are derived largely from expert opinion and mirror the advice surrounding autoHCT.

#### 3.3.1 Vaccinations prior to CAR‐T

As demonstrated by the seroprevalence surveys, most patients who demonstrated humoral antibody response to vaccine‐specific pathogens prior to CAR‐T, maintained seropositivity at follow‐up.^110^, ^111^, ^116^ Optimizing timely revaccination following autoHCT or HCT might potentially improve seroprotection in the event of relapse and subsequent need for CAR‐T therapies.

Considering the epidemic nature of both influenza and SARS‐CoV‐2, the EBMT/JACIE/EHA and ASH‐ASTCT consensus guidelines advocate for vaccination against SARS‐CoV‐2 and influenza at least 2 weeks prior to lymphodepletion, citing the likely lack of vaccine response after CAR‐T infusion due to B‐cell aplasia.^75^, ^138^ Expert commentary has extended this recommendation to include pre‐CAR‐T vaccination against *S. pneumoniae* and hepatitis B in high endemicity areas and consideration of vaccination of household contacts.^100^

#### 3.3.2 Vaccinations following CAR‐T

Vaccine selection and dosing intervals for CAR‐T patients mimic guidelines for HCT summarized in Table 1. Revaccination post‐CAR‐T is derived from expert consensus, center and physician opinion, with general recommendations summarized in Table 3 and an example vaccination schedule in Table 4.^99^

**TABLE 3:** Recommendations on revaccination timing post cellular therapy from expert international guidelines, expert center protocols, and expert opinion.

	Consensus guidelines (EBMT/JACIE/EHA, ASH‐ASTCT)	Expert center protocols	Expert opinions
Killed and inactivated vaccines: PCV13 (3 doses) PPSV23 (1 doses) DTaP (3 doses) HBV (3 doses) HAV (2 doses) SARS‐CoV‐2 (3+ doses) IIV (2 doses) VZV (aRZV) (2 doses) ACYW‐135 (2 doses)^a^ Hib (3 doses)^a^	≥3 months after CAR‐T therapy AND Demonstrated immune reconstitution^*^ AND No ongoing immunosuppression (see text).	No IVIg in prior 2 months No anti‐CD19 or anti‐CD20 antibody therapy in prior 6 months Demonstrate vaccine response on one killed vaccine prior to revaccination with remaining schedule	Demonstrate detectable serum IgA (evidence of class switching) If no vaccine response demonstrated, re‐check immune reconstitution in 6 months and continue IVIg
Live vaccines MMR (2 doses) VZV (LAVV) (2 doses) Many travel vaccines (e.g., typhoid, yellow fever, and polio oral vaccine)	≥12 months after CAR‐T therapy AND Demonstrated immune reconstitution^*^ AND No ongoing immunosuppression AND No IVIg in prior 8 months	No IVIg in prior 5 months No anti‐CD19 or anti‐CD20 antibody therapy in prior 6 months No HCT ≤2 years prior No systemic immunosuppression in prior 12 months Demonstrated response to killed/inactive vaccine	Demonstrate detectable serum IgA (evidence of class switching)

Abbreviations: ACYW‐135, meningococcal quadrivalent conjugate vaccine; aRZV, adjuvanted recombinant zoster virus vaccine; CAR‐T, chimeric antigen receptor T cell; CD19, cluster of differentiation 19; CD20, cluster of differentiation 20; DTaP, higher dose diphtheria, tetanus, and acellular pertussis vaccine; HAV vaccine, hepatitis A virus; HBV vaccine, hepatitis B virus; HCT, hematopoietic cell transplant; Hib, *Haemophilus influenzae* type B conjugate vaccine; IgA, immunoglobulin A; IIV, inactivated influenza vaccine; IVIg, intravenous immunoglobulin; LAVV, live‐attenuated varicella vaccine; MMR, measles, mumps, and rubella live virus vaccine; PCV, pneumococcal conjugate vaccine; PPSV23, pneumococcal 23‐valent polysaccharide vaccine; SARS‐CoV‐2, severe acute respiratory syndrome coronavirus 2; VZV, varicella zoster virus.

^a^ Revaccination against meningococcal and hemophilus to be considered in patients with CAR‐T patients and additional risk factors, such as splenectomy.

**TABLE 4:** Vaccination schedule^a^ for adult patients treated with chimeric antigen receptor T‐cell (CAR‐T) therapy.^99^

Vaccines	Pre‐CAR‐T	≥6 months	≥8 months	≥10 months	≥12 months	≥18 months
IIV	Influenza	Influenza				
PCV		PCV13	PCV13	PCV13		
PPSV23						PPSV23
DTaP		DTaP	Td	Td		
HAV		HAV			HAV	
HBV		HBV	HBV		HBV	
Varicella zoster^b^					aRZV	aRZV

Abbreviations: aRZV, adjuvant recombinant zoster vaccine; DTaP, higher dose diphtheria, tetanus, and acellular pertussis vaccine; HAV vaccine, hepatitis A virus; HBV vaccine, hepatitis B virus; IIV, inactivated influenza vaccine; PCV, pneumococcal conjugate vaccine; PPSV23, pneumococcal 23‐valent polysaccharide vaccine; Td, tetanus–diphtheria.

^a^ This schedule was published prior to the availability of coronavirus disease 2019 (COVID‐19) vaccines. Revaccination with a primary course of three mRNA COVID‐19 vaccines is now recommended for patients following CAR‐T therapy.^75^

^b^ Seropositive patients.

Consensus guidelines advise that killed or inactivated vaccines be administered ≥3 months after CAR‐T administration. Patients should demonstrate immune reconstitution prior to vaccination, defined as CD4+ >0.2 × 10^9^/L, CD19 or CD20+ B cells >0.2 × 10^9^/L.^99^, ^138^, ^139^ Patients should not be receiving concomitant immunosuppressive treatment including cytotoxic chemotherapy, systemic corticosteroids, T‐cell‐depleting or anti‐lymphocyte agents, or IVIg within the previous 2 months.^99^, ^138^, ^139^ Certain immunotherapies may not be considered additionally immunosuppressive in the context of inactivated vaccines, such as checkpoint inhibitors, or immunomodulatory agents.^139^ In patients with multi‐modal immunomodulatory therapy, expert infectious disease consultation is advised.^11^, ^99^, ^140^ The exception is the influenza vaccine, which should be given annually prior to the expected influenza season, and ideally 2 weeks prior to lymphodepleting chemotherapy.^93^

Live vaccines should be different until at least 1 year following CAR‐T and require demonstration of immune reconstitution. Several expert groups suggest that live vaccines are contraindicated within 8 months of receiving IVIg replacement.^138^ Expert centers, for example, the Fred Hutchinson Cancer Center, recommend delaying live and non‐live adjuvant vaccines until ≥5 months from last IVIg replacement.^139^ Further expert commentary has suggested that before live and non‐live adjuvant vaccines, vaccine responses to killed/attenuated vaccines should be demonstrated.^99^, ^139^

#### 3.3.3 Future directions

For HCT recipients, owing to differences in conditioning regimen, stem cell source, and underlying disease, there is an opportunity to refine and personalize revaccination schedules for autologous HCT recipients rather than extrapolating from allogeneic HCT recipients. Ongoing assessment by clinical studies is important for newer vaccine formulations, including PCV20, aRZV, and the bivalent COVID‐19 vaccines. Alternate vaccination strategies to improve immunogenicity, such as examining new dosing schedules of vaccination, are required. Candidate RSV vaccines^22^ are in development, going forward, it will be important to advocate for the inclusion of HCT recipients in clinical trials to understand their safety and efficacy.

Significantly more research is required to optimize the vaccine schedule for cellular therapy (CAR‐T) treated patients. Longitudinal, prospective observational studies are required to properly assess retention of antibody‐specific IgG from baseline, through treatment, and following immune reconstitution to determine which vaccines, if any, are most required. Vaccine response in BCMA CAR‐T treated patients also warrants investigation. Clinical trials are required to determine the best way to time vaccine initiation post‐CAR‐T, directly comparing standard timepoints to timepoints driven by markers of immune reconstitution, or following demonstrable vaccine response. Methods to heighten immunogenicity, including booster regimens, should also be explored.

## 4 CONCLUSION

In summary, there are well‐recognized vaccine schedules for the HCT population, with new vaccine formulations including PCV20, aRZV, and bivalent booster COVID‐19 vaccines that require ongoing assessment. Revaccination post‐cellular therapies at present mostly follow the HCT schedule, but further research is required to optimize the timing of vaccine delivery and clarify which vaccines would be of most benefit to the cellular therapy patients.

## CONFLICT OF INTEREST STATEMENT

Victoria G. Hall and Gemma Reynolds have no conflicts of interest. Benjamin W. Teh has been on the advisory board for Moderna, CSL‐Behring, and Takeda. He has also received research funding from Merck Sharp and Dohme, Sanofi, and Seqirus.

## Data Availability

This is a review article with no new original data provided.
